# Supplementary material for: Ant abaecin-2 is a context-dependent copper-binding effector that can be either inhibitory or protective
Source: RSC Chem Biol. 2026 Jul 20. Online ahead of print. doi: 10.1039/d6cb00146g (PMC13398410; doi:10.1039/d6cb00146g)
Supplement: CB-OLF-D6CB00146G-s001 [file CB-OLF-D6CB00146G-s001.pdf]

Supplementary Information (SI) for RSC Chemical Biology.
This journal is © The Royal Society of Chemistry 2026

Supporting Material for

Ant abaecin-2 is a context-dependent copper-binding effector that can be either inhibitory or protective

Caroline M. Donaghy[1], Helena Heyer-Gray[2], Charlotte O'Hern[3], Michael Ibrahim[1], Bryant Perez-Torres[1], Nichali Bogues[1], Andrei T. Alexandrescu[2], Karrera Djoko[3], Jonathan L. Klassen[2,4], Alfredo M. Angeles-Boza[1]

1. Department of Chemistry, University of Connecticut, Storrs, Connecticut, USA
2. Department of Molecular and Cell Biology, University of Connecticut, Storrs, Connecticut, USA
3. Durham University, Department of Biosciences, Durham, UK
4. Institute for Systems Genomics, University of Connecticut, Storrs, Connecticut, USA

<u>**Table of Contents:**</u>

Figure S1: Maximum likelihood tree of ant abaecin-2 protein sequences.
Figure S2: Maximum likelihood tree of ant abaecin-2 nucleotide sequences.
Figure S3. Mass spectrometry of abaecin-2 peptides to determine their copper-binding ability.
Figure S4: Copper recovery from *A. colombica* abaecin-2 after desalting.
Figure S5: Cu(II) binding affinity of *A. colombica* abaecin-2.
Figure S6: 1D NMR spectra of abaecin-2 WT and H3 illustrating Cu(II) induced line broadening.
Figure S7: NMR broadening of WT-abaecin-2 TOCSY cross-peaks with Cu(II) affects primarily the first nine residues in the sequence.
Figure S8: Superposition of NMR spectra illustrating differences due to the H3A mutation between WT-abaecin-2 and H3A-abaecin-2.
Figure S9: Multiplicity-edited ^{13}C-HSQC NMR spectra illustrating signal broadening due to Cu(II) in WT-abaecin-2 and H3A-abaecin-2.
Figure S10. Comparison of random coil index (RCI) values for abaecin-2 WT and the H3A mutant.
Figure S11. Interaction of *A. texana* abaecin-2 with LPS
Figure S12. Differences in the rescuing effect of *A. texana* WT and H3A abaecin-2 peptides.
Table S1: Abaecin-2 sequences used for BLASTp searches.
Table S2: Hymenopteran abaecin sequence data returned by BLAST searches.
Table S3: ICP analysis sample metadata.
Table S4. Susceptibility of *E. coli* JW 0473-3 (Δ*copA*) to *A. texana* and *A. colombica* abaecin-2 variants in combination with the pore-forming peptide cecropin A.
Supplemental code S1. Sample DynaFit scripts used for Cu(II) binding affinity determination of abaecin-2 peptides.

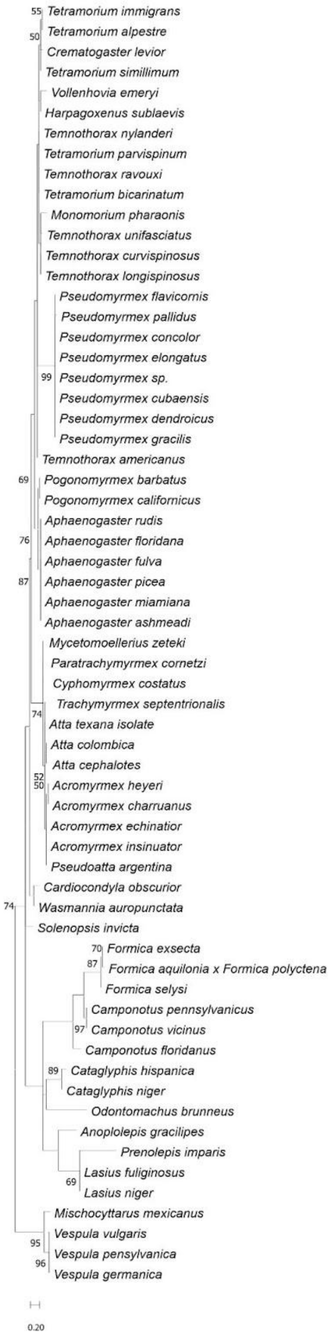

Figure S1. Maximum likelihood tree of ant abaecin-2 protein sequences. Protein phylogeny generated from abaecin-2 peptide sequences using the MEGA JTT+G substitution model with 500 bootstrap replicates. Abaecin-2 sequences from 4 wasp species (*Mischocyttarus mexicanus, Vespula vulgaris, V. pensylvanica,* and *V. germanica*) were used as an outgroup to root the tree. Local bootstrap values > 50 are shown. Scale bar corresponds to the number of amino acid substitutions per site.

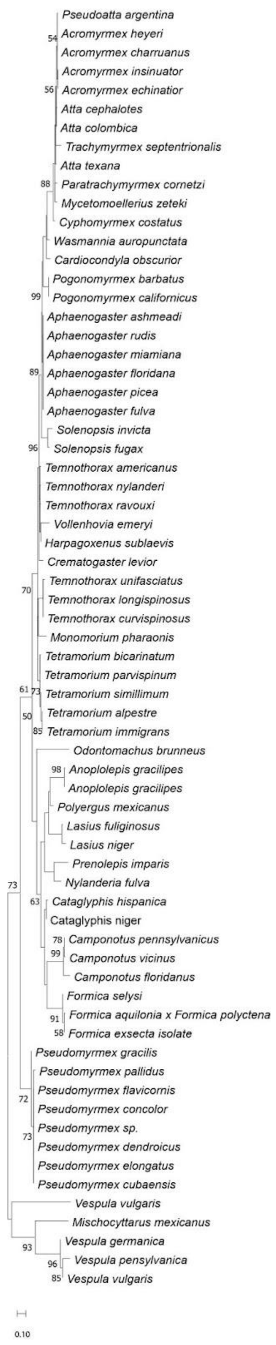

Figure S2. Maximum likelihood tree of ant abaecin-2 nucleotide sequences. Nucleotide phylogeny generated from abaecin-2 coding nucleotide sequences using the MEGA K2 substitution model with 500 bootstrap replicates. Abaecin-2 nucleotide sequences from 4 wasp species (*Mischocyttarus mexicanus, Vespula vulgaris, V. pensylvanica,* and *V. germanica*) were used as an outgroup to root the tree. Local bootstrap values > 50 are shown. Scale bar corresponds to the number of nucleotide substitutions per site.

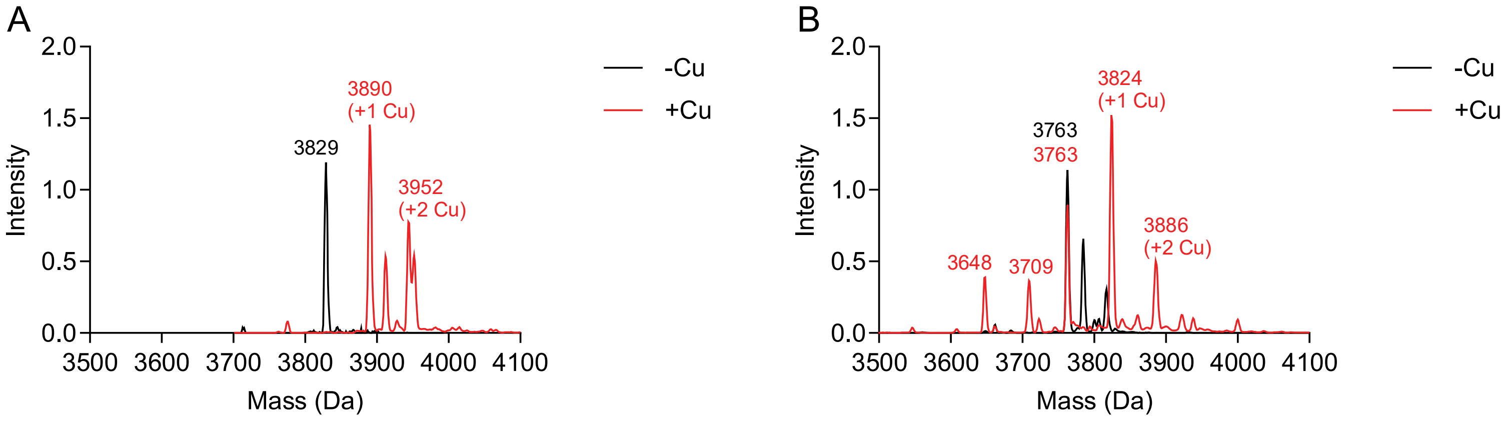

Figure S3. Mass spectrometry of abaecin-2 peptides to determine their copper-binding ability. (A) Wildtype (WT) *A. texana* abaecin-2 incubated in the presence or absence of 5 molar equivalents of Cu(II). (B) H3A *A. texana* abaecin-2 incubated in the presence or absence of 5 molar equivalents of Cu(II). Both peptides can bind up to two Cu(II) ions, but binding is weaker in the H3A mutant.

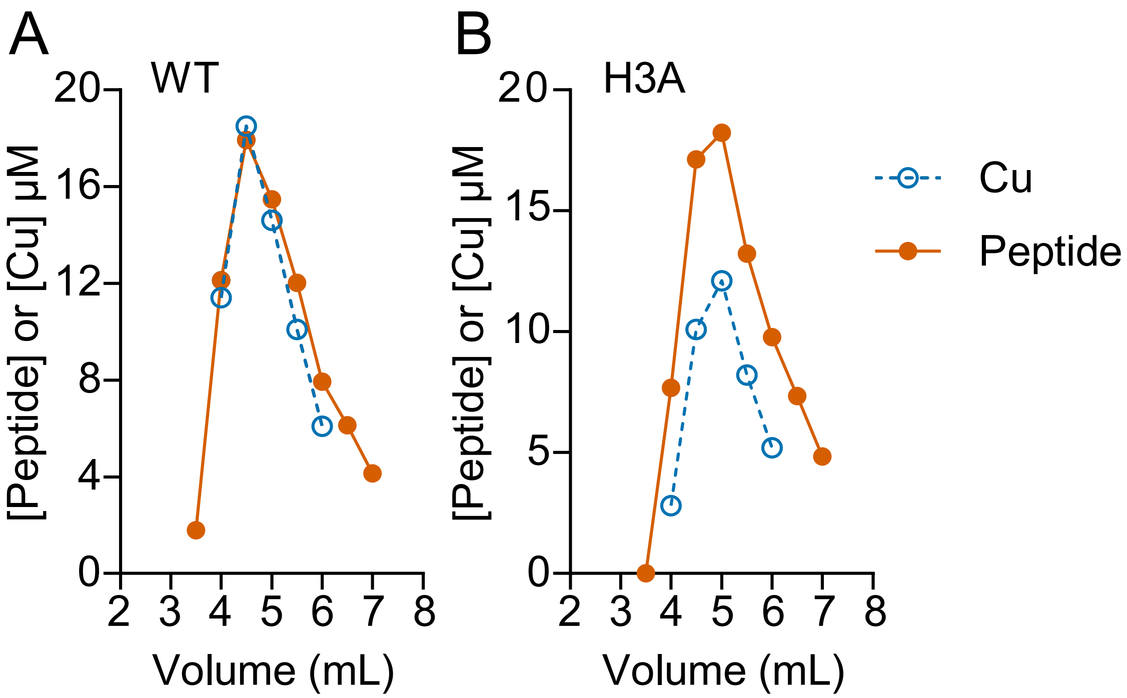

Figure S4. Copper recovery from *A. colombica* abaecin-2 after desalting. Elution of WT (A) and H3A (B) abaecin-2 peptides from PD-10 desalting column. Data points show the concentrations of peptide (filled circles, ●) and Cu(II) ions (open circles, ○) in each fraction. Data are from one representative of three independent experiments.

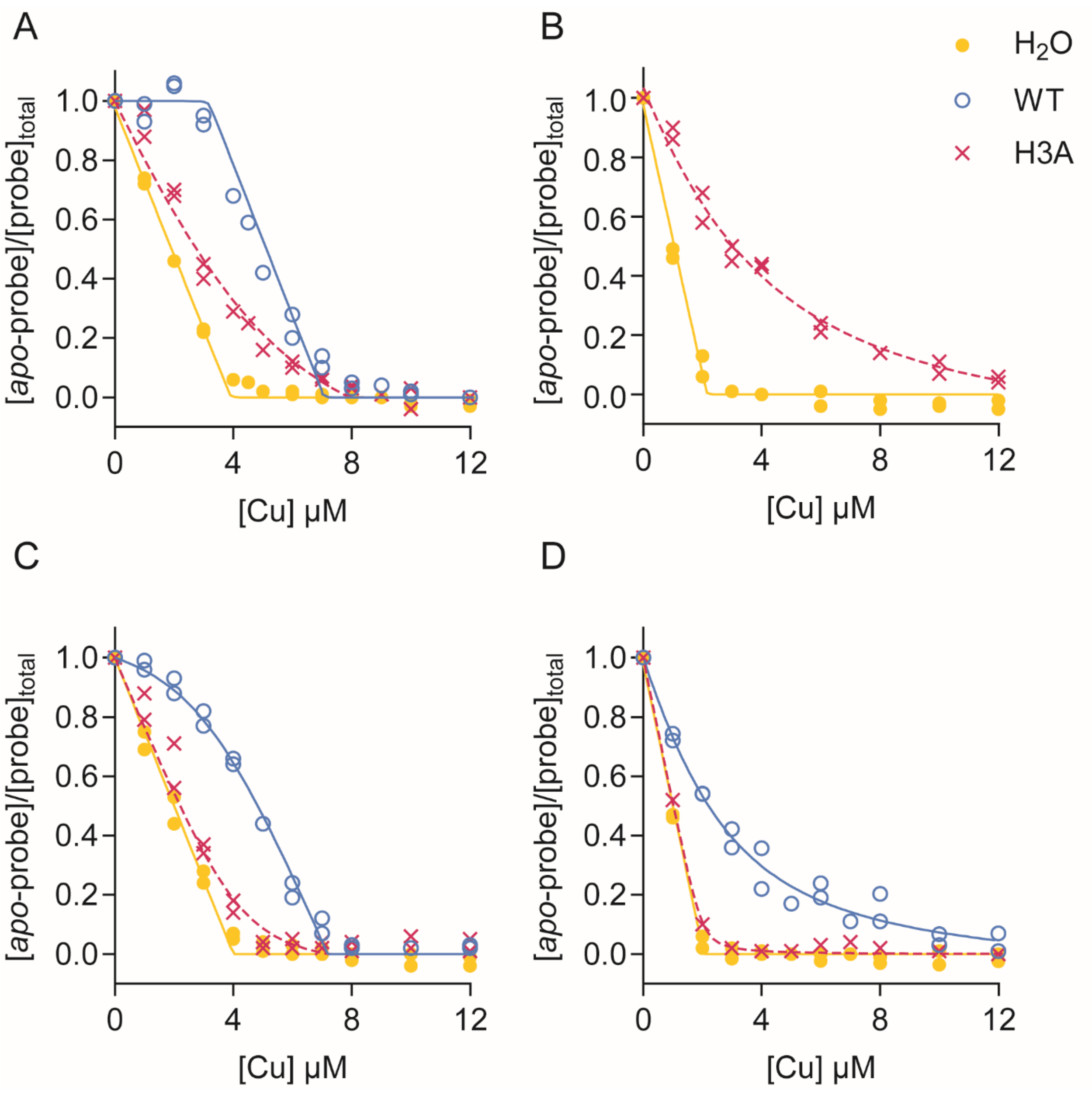

Figure S5. Cu(II) binding affinity of *A. colombica* abaecin-2. The WT (○, solid lines) or H3A (x, dashed lines) peptides were competed against the fluorescence probe DP2, DP3, or DP4. Data points (n = 2) show the resulting competition curves when Cu(II) was added to mixtures of (A) 4 μM DP2 and 3.2 μM peptide, (B) 2 μM DP2 and 12.8 μM peptide, (C) 4 μM DP3 and 3.2 μM peptide, or (D) 2 μM DP4 and peptide. Control curves without any peptide are also shown (●, solid lines).

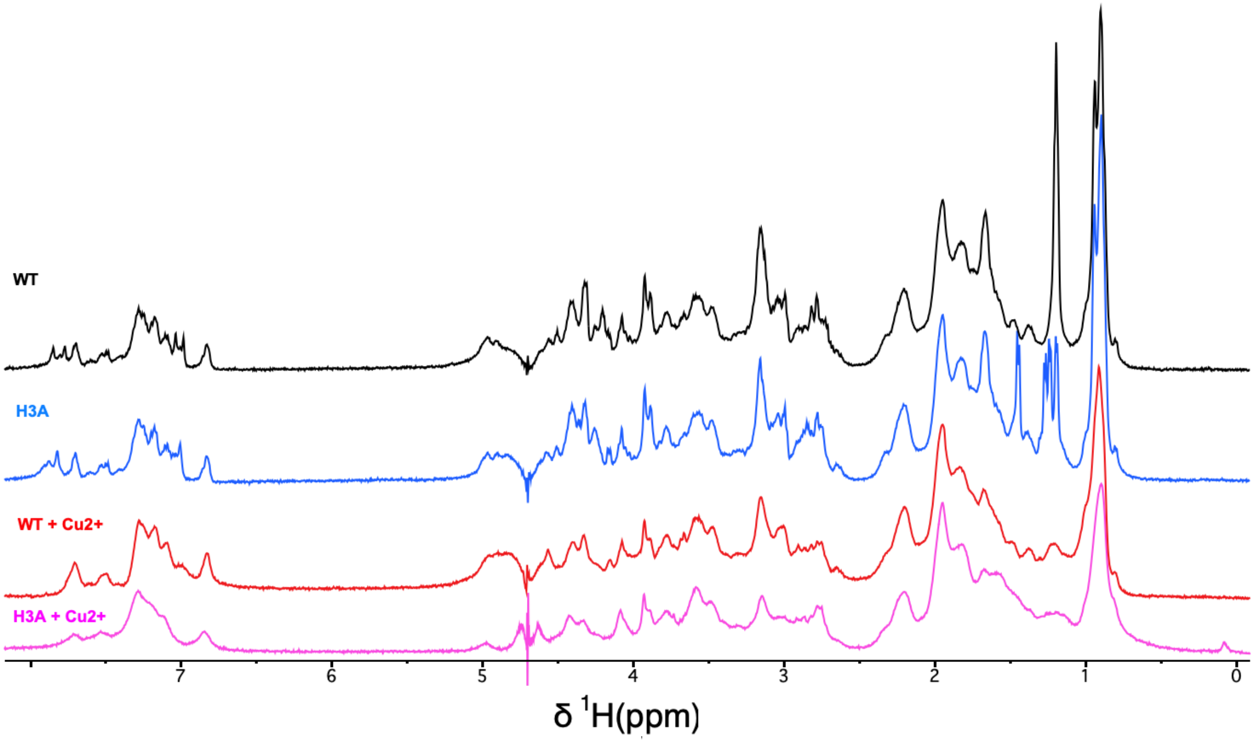

Figure S6: 1D NMR spectra of abaecin-2 WT and H3A illustrating Cu(II) induced line broadening. WT and H3A concentrations were 0.5 mM in the presence or absence of 0.6 mM $CuCl_2$ (1.2 molar equivalents to abaecin-2). Data were collected at 500 MHz and a temperature of 32 °C for samples in D_2O, with a pD of 6.6 ± 0.2.

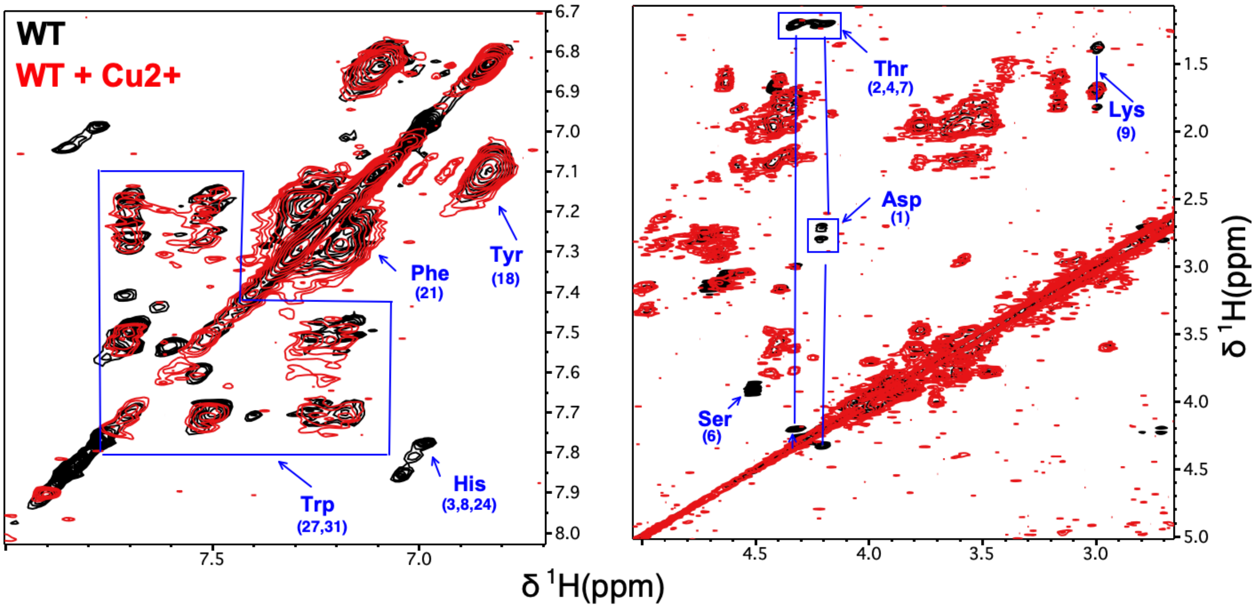

Figure S7: NMR broadening of WT-abaecin-2 TOCSY cross-peaks with Cu(II) affects primarily the first nine residues in the sequence. Superposition of 70 ms mixing time 2D TOCSY spectra for WT-abaecin-2 in the presence (red) or absence (black) of 1.2 molar equivalents of $CuCl_2$. Left and right panels show the aromatic and aliphatic portions of the spectra. Most of the spin systems affected by Cu(II) are in the first nine residues (sequence numbers are given in parentheses), whereas the other cross-peaks are largely unaffected. The exceptions are the Hε1-Hδ2 cross-peaks of H24, and two sidechain cross-peaks from either W27 or W31 that are also absent in the presence of Cu(II). TOCSY spectra were recorded at 500 MHz on a 0.5 mM WT-abaecin-2 sample at pD 6.6.

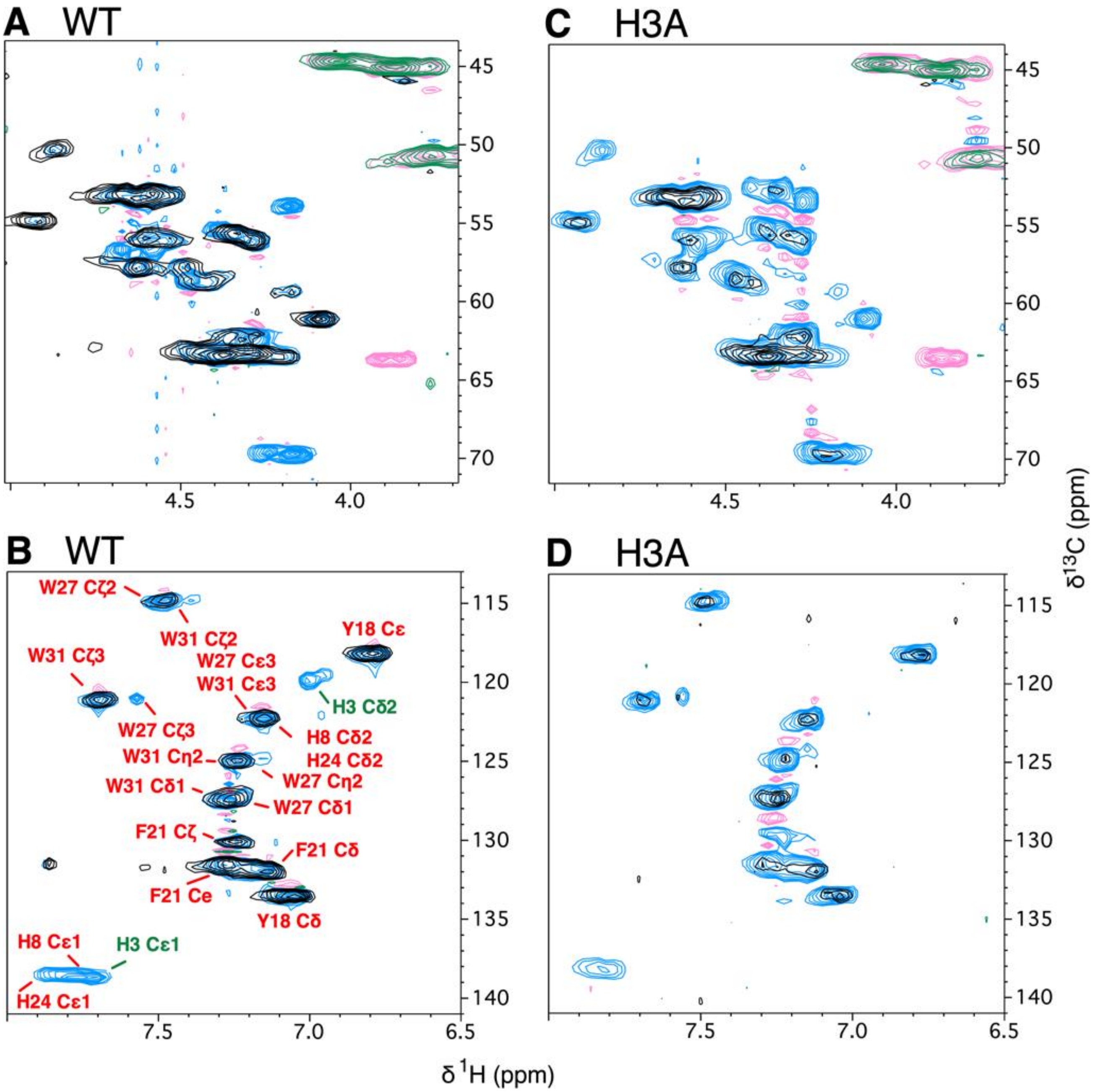

Figure S8: Multiplicity-edited ^{13}C-HSQC NMR spectra illustrating NMR signal broadening due to Cu(II) in abaecein-2 WT and the H3A mutant. In the multiplicity-edited ^{13}C-HSQC experiments, CH and CH_3 groups have positive contours while CH_2 groups have negative contours. The Hα-Cα fingerprint regions and aromatic regions of the spectrum respectively are shown for WT (**A**, **B**) and H3A (**C**, **D**). For the spectra in the absence of Cu(II), contour levels are shown in blue (positive) and pink (negative). The spectra in the presence of Cu(II) are shown with black (positive) and green (negative) contours. For all panels, the spectra in the presence of Cu(II) are shown at twice the amplitude to emphasize the line broadening. The assignments for the Hα-Cα region are given in Figure 7B of the main text. The assignments for the aromatic region are given in panel B of this figure. The multiplicity-edited ^{13}C spectra were collected at 800 MHz at a temperature of 10 °C. WT

and H3A concentrations were 1 mM in the presence or absence of 1.2 mM $CuCl_2$ (1.2 molar equivalents to abaecin-2). Note that Cu(II)-induced NMR broadening is more widespread in H3A and more specific in WT.

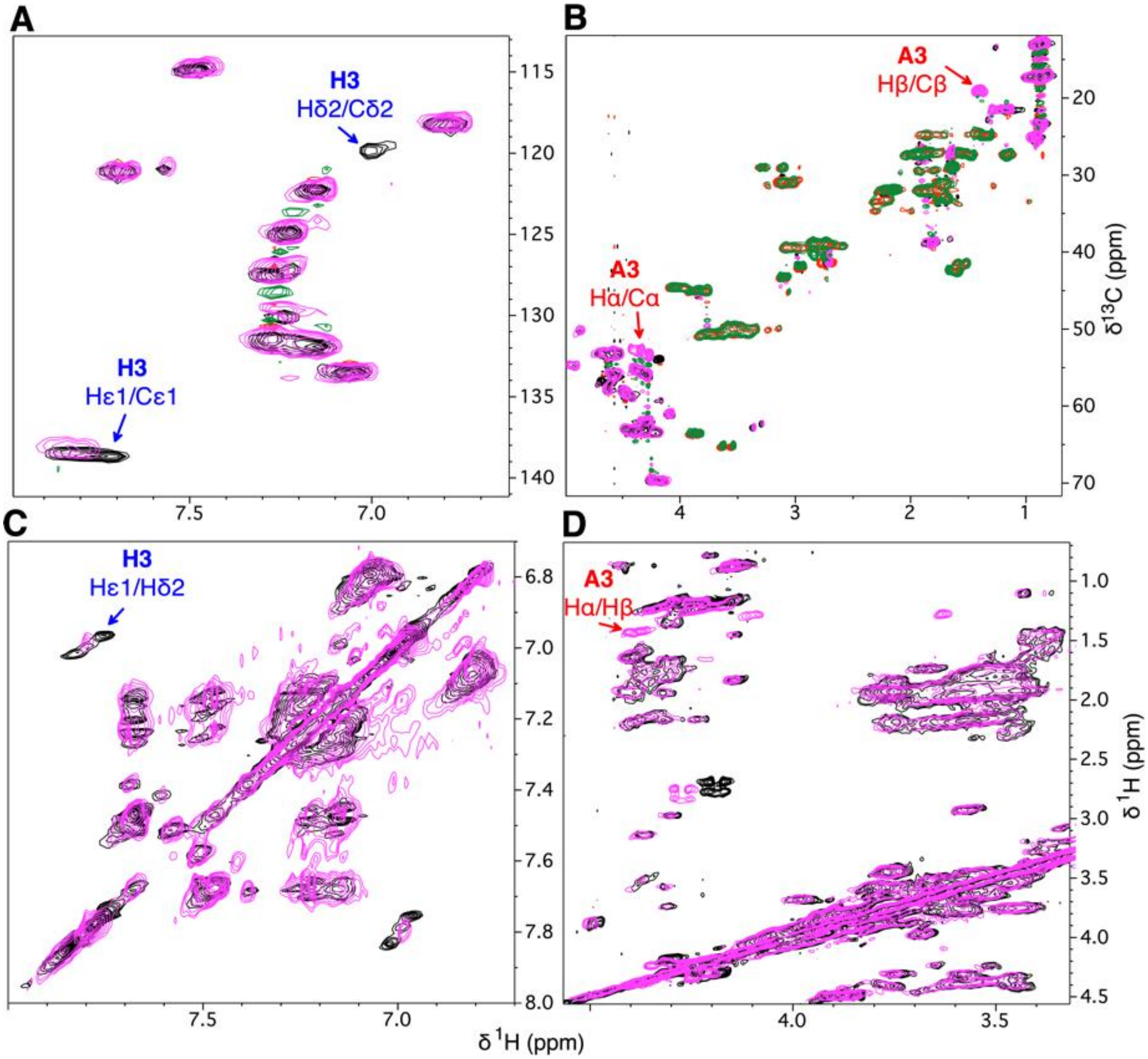

Figure S9: Superposition of NMR spectra illustrating differences due to the H3A mutation in abaecin-2. Aromatic (**A**) and aliphatic (**B**) regions of the superposed multiplicity-edited ^{13}C-HSQC spectra of WT (black for positive contours from CH and CH_3 groups, red for negative contours from CH_2 groups) and H3A (pink for positive contours and green for negative contours). The extra black contours due to the Hε1/Cε1 and Hδ2/Cδ2 cross-peaks of H3 in WT compared to H3A are labeled blue in (A). Similarly, the extra pink contours due to the Hα/Cα and Hβ/Cβ cross-peaks of A3 in H3A compared to WT are labeled red in (B). The bottom two panels show superposed aromatic (**C**) and aliphatic (**D**) regions from the 2D TOCSY spectra for WT (black) and H3A (pink). The differences between the two spectra due to the presence of H3 in WT and A3 in H3A, are labeled blue and red, respectively. The multiplicity-edited ^{13}C-HSQC spectra were collected at 800 MHz using 1 mM samples at a temperature of 10 °C. The TOCSY data were collected at 500 MHz using 0.5 mM samples at a temperature of 32 °C.

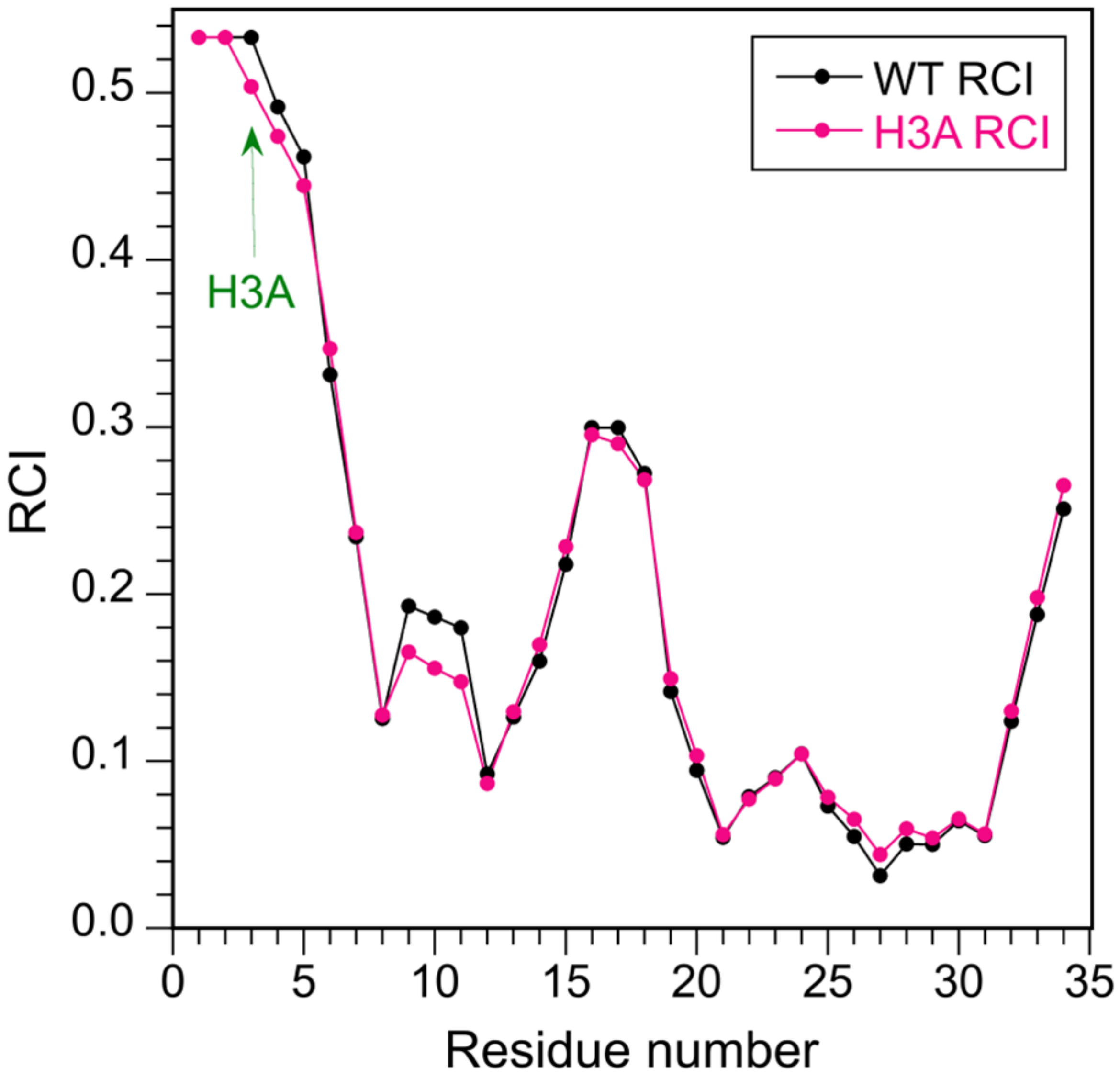

Figure S10. Comparison of random coil index (RCI) values for abaecin-2 WT and the H3A mutant. The site of the H3A mutation is denoted with a green arrow in the plot. The RCI sequence profiles are very close for the WT and mutant, consistent with their ^{1}H-^{13}C HSQC and TOCSY spectra being superposable (Fig. S9), and indicate similar levels of disorder.

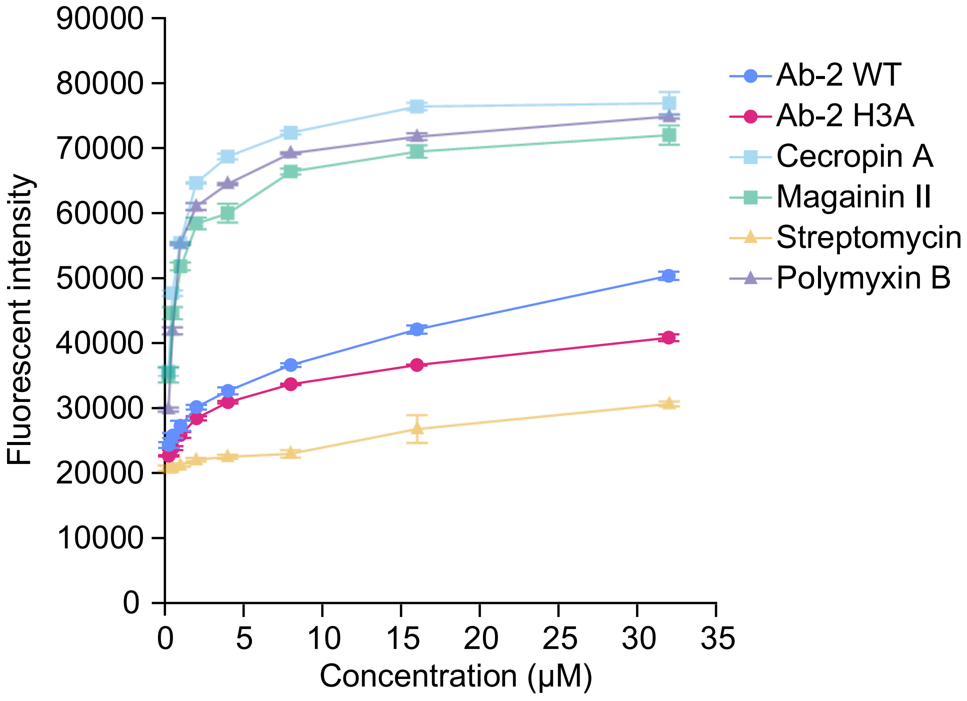

Figure S11. LPS binding by abaecin-2 peptides, pore-forming peptides, and antibiotic controls. Data points show the average (± SE from 2 replicates) fluorescent intensity observed during BODIPY-TR-cadaverine displacement assays. Both WT and H3A abaecin-2 peptides interact only moderately with LPS, unlike magainin-2, cecropin A, and polymyxin B.

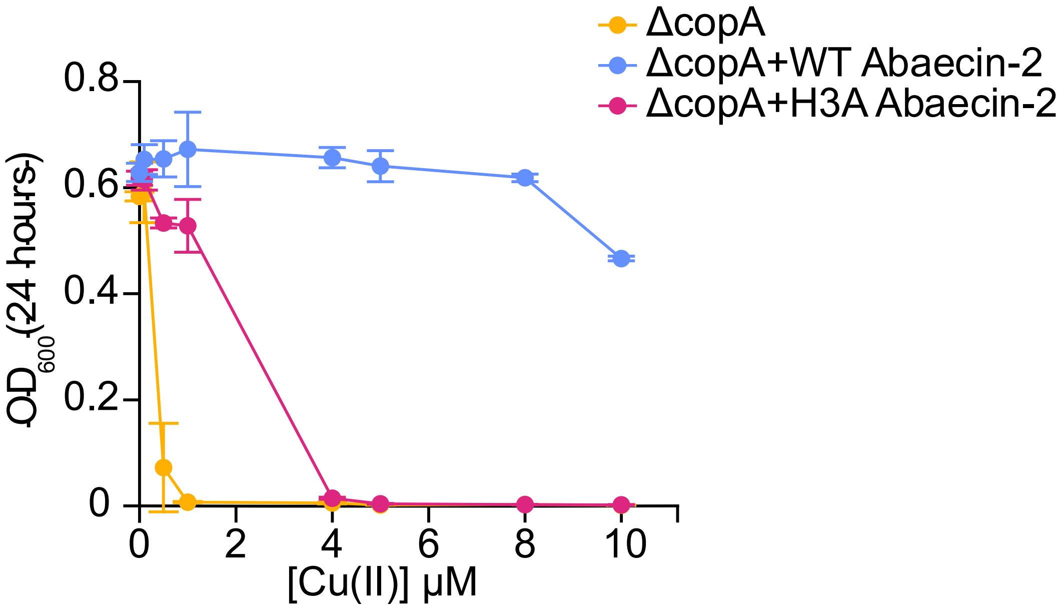

Figure S12. Differences in the rescuing effect of *A. texana* WT and H3A abaecin-2 peptides. Data points show growth of either the Δ*copA* mutant strain of *E. coli*, represented by the Optical Density at 600 nm after 24 h incubation at 37 °C, in the presence of Cu(II), with or without 10 μM of WT or H3A *A. texana* abaecin-2.

Table S1. Abaecin-2 sequences used for BLASTp searches.

**Species**	**Abaecin-2 sequence**
A. colombica	<u>DTH</u>TFSTHKIRLPNGPGYGPFNPHQPWPIPWPNG
A. texana	<u>DTH</u>TLSTHKIRLPNGPGYGPFNPHQPWPIPWPNG
Mycetomoellerius zeteki	MRFTMYIVMLLVAICALFA<u>DTH</u>TLLTHRIRLPNGPGYGPFNPHQPWPIPWPNNG
Acromyrmex echinatior	MRLTMYIVMLLITICALFA<u>DIH</u>TLSTHKIRLPNGPGYGPFNPHQPWPIPWPNNG
Wasmannia auropunctata	MKFAMYMVMLLVAICALFA<u>DTH</u>ALPKHRFRLPSGPGYGPFNPQQPWPVPWPNNG
Pseudomyrmex gracilis	FKNNTHIVNMKFAIYAVMLLVAICALFANTQALPGRRSSLPCGPGYGPFNPRKSWPIPLPKHG
Pogonomyrmex barbatus	MRFSFPITTSFISLQSNIFNMRFAAYIVTLLVAICALFA<u>DTH</u>ALPKHRLRLPGGGPGYGPFNPRLPWPIPLPNRDH
Cyphomyrmex costatus	FYFPVTIFFDQNNIFNMRLRFTMYIVMLLVAICALFV<u>DTH</u>TLSTHRIRLPSGPGYGPFNPHQPWLISWPNQ
Paratrachymyrmex cornetzi	MRFTMYIVMLLIATCALFV<u>DTH</u>TLSTHRIRLPNGPGYGPFNPHQSWPIPLPNNG

Table S2. Hymenopteran abaecin sequence data returned by BLAST searches.

Name	Organism	Accession number	Contig position	Abaecin type	Search method	Annotated gene model present?*
Pogonomyrmex barbatus	Ant	XP 025074443.1	1-49	Abaecin-1	PSI-BLAST Search Result	No
Acromyrmex echinatior	Ant	EGI68768.1	1-51	Abaecin-1	PSI-BLAST Search Result	No
Atta colombica	Ant	KYM77085.1	1-51	Abaecin-1	PSI-BLAST Search Result	No
Cyphomyrmex costatus	Ant	KYM99866.1	1-58	Abaecin-1	PSI-BLAST Search Result	No
Harpegnathos saltator	Ant	EFN88303.1	49-97	Abaecin-1	Translated BLASTn Search Result	No
Mycetomoellerius zeteki	Ant	KYQ52474.1	1-51	Abaecin-1	PSI-BLAST Search Result	No
Ooceraea biroi	Ant	EZA47299.1	1-52	Abaecin-1	Translated BLASTn Search Result	No
Paratrachymyrmex cornetzi	Ant	KYN17873.1	1-51	Abaecin-1	PSI-BLAST Search Result	No
Solenopsis invicta	Ant	AFJ94682.1	1-51	Abaecin-1	Translated BLASTn Search Result	No
Temnothorax curvispinosus	Ant	XP 024869487.1	1-52	Abaecin-1	Translated BLASTn Search Result	No
Apis cerana japonica	Bee	BAI58048.1	1-53	Abaecin-1	Translated BLASTn Search Result	No
Apis cerana	Bee	XP 016919244.1	1-53	Abaecin-1	Translated BLASTn Search Result	No
Apis cerana cerana	Bee	ACH90419.1	1-53	Abaecin-1	Translated BLASTn Search Result	No
Apis florea	Bee	XP 003699027.2	21-78	Abaecin-1	Translated BLASTn Search Result	No
Apis mellifera	Bee	NP 001011617.1	1-53	Abaecin-1	Translated BLASTn Search Result	No
Bombus affinis	Bee	XP 050578474.1	22-85	Abaecin-1	Translated BLASTn Search Result	No
Bombus ignitus	Bee	AAQ90411.1	1-58	Abaecin-1	Translated BLASTn Search Result	No
Bombus impatiens	Bee	XP 003491544.1	1-58	Abaecin-1	Translated BLASTn Search Result	No
Bombus pyrosoma	Bee	XP 043604743.1	45-116	Abaecin-1	Translated BLASTn Search Result	No
Bombus terrestris	Bee	XP 003394701.1	22-85	Abaecin-1	Translated BLASTn Search Result	No
Bombus vancouverensis nearcticus	Bee	XP 033205111.1	22-85	Abaecin-1	Translated BLASTn Search Result	No
Ceratina calcarata	Bee	XP 017876439.1	1-55	Abaecin-1	Translated BLASTn Search Result	No
Eufriesea mexicana	Bee	XP 017760992.1	1-51	Abaecin-1	Translated BLASTn Search Result	No
Frieseomelitta varia	Bee	XP 043524279.1	1-54	Abaecin-1	Translated BLASTn Search Result	No
Habropoda laboriosa	Bee	XP 017790090.1	1-56	Abaecin-1	Translated BLASTn Search Result	No
Heterotrigona itama	Bee	CAD1479620.1	1-61	Abaecin-1	Translated BLASTn Search Result	No
Melipona bicolor	Bee	KAK1128096.1	1-50	Abaecin-1	Translated BLASTn Search Result	No
Melipona quadrifasciata	Bee	KOX74422.1	1-52	Abaecin-1	Translated BLASTn Search Result	No
Athalia rosae	Sawfly	XP 012260680.2	1-51	Abaecin-1	Translated BLASTn Search Result	No

Belonocnema kinseyi	Wasp	XP 033225186.1	2-67	Abaecin-1	Translated BLASTn Search Result	No
Copidosoma floridanum	Wasp	XP 014208617.1	16-49	Abaecin-1	Translated BLASTn Search Result	No
Fopius arisanus	Wasp	XP 011309145.1	1-55	Abaecin-1	Translated BLASTn Search Result	No
Leptopilina boulardi	Wasp	XP 051165230.1	1-54	Abaecin-1	Translated BLASTn Search Result	No
Leptopilina heterotoma	Wasp	XP 043474498.1	1-53	Abaecin-1	Translated BLASTn Search Result	No
Nasonia vitripennis	Wasp	XP 003425826.1	1-59	Abaecin-1	Translated BLASTn Search Result	No
Phymastichus coffea	Wasp	XP 058790769.1	15-55	Abaecin-1	Translated BLASTn Search Result	No
Pteromalus puparum	Wasp	ABS44869.1	1-51	Abaecin-1	Translated BLASTn Search Result	No
Trichomalopsis sarcophagae	Wasp	OXU21302.1	34-67	Abaecin-1	Translated BLASTn Search Result	No
Vespa crabro	Wasp	XP 046820244.1	116-184	Abaecin-1	Translated BLASTn Search Result	No
Vespula germanica	Wasp	KAF7405906.1	1-56	Abaecin-1	Translated BLASTn Search Result	No
Vespula pensylvanica	Wasp	KAF7429375.1	55-117	Abaecin-1	Translated BLASTn Search Result	No
Vespula vulgaris	Wasp	KAF7402542.1	55-120	Abaecin-1	Translated BLASTn Search Result	No
Atta texana	Ant	QEPB01039057.1	6713-6811	Abaecin-2	Translated BLASTn Search Result	No
Crematogaster levior	Ant	CABEGB010000914.1	269983 -270081	Abaecin-2	Translated BLASTn Search Result	No
Temnothorax nylanderi	Ant	JASTWM010000507.1	4649-4747	Abaecin-2	Translated BLASTn Search Result	No
Acromyrmex charruanus	Ant	JAANIC010005180.1	528137 -528235	Abaecin-2	Translated BLASTn Search Result	No
Acromyrmex echinatior	Ant	XP 011049187.1	1-54	Abaecin-2	PSI-BLAST Search Result	Yes
Acromyrmex echinatior	Ant	JANHNX010000014.1	2396933-2397031	Abaecin-2	Translated BLASTn Search Result	Yes
Acromyrmex heyeri	Ant	JAANIB010010238.1	325671-325769	Abaecin-2	Translated BLASTn Search Result	No
Acromyrmex insinuator	Ant	JAANHZ010000870.1	103064 -103162	Abaecin-2	Translated BLASTn Search Result	No
Anoplolepis gracilipes	Ant	JAOBUE010000016.1	3450394 -3450555	Abaecin-2	Translated BLASTn Search Result	No
Aphaenogaster ashmeadi	Ant	NJRQ01000102.1	140396-140575	Abaecin-2	Translated BLASTn Search Result	No
Aphaenogaster floridana	Ant	NJRP01000116.1	328977-329201	Abaecin-2	Translated BLASTn Search Result	No
Aphaenogaster fulva	Ant	NJRO01000023.1	759564-759788	Abaecin-2	Translated BLASTn Search Result	No
Aphaenogaster miamiana	Ant	NJRN01000078.1	355578-355802	Abaecin-2	Translated BLASTn Search Result	No
Aphaenogaster picea	Ant	NJRM01000023.1	444152-444376	Abaecin-2	Translated BLASTn Search Result	No
Aphaenogaster rudis	Ant	NJRK01000139.1	123953-124177	Abaecin-2	Translated BLASTn Search Result	No
Atta cephalotes	Ant	ADTU01009315.1	9729-9827	Abaecin-2	Translated BLASTn Search Result	No
Atta colombica	Ant	LKEW01028451.1	1134 -1286	Abaecin-2	Translated BLASTn Search Result	No
Atta colombica	Ant	KYM77084.1	1-43	Abaecin-2	PSI-BLAST Search Result	No
Camponotus floridanus	Ant	AEAB01017815.1	1092-1181	Abaecin-2	Translated BLASTn Search Result	No

Camponotus pennsylvanicus	Ant	JALHBL010000095.1	290527-290679	Abaecin-2	Translated BLASTn Search Result	No
Camponotus vicinus	Ant	JANXEZ010000009.1	13828362 -13828514	Abaecin-2	Translated BLASTn Search Result	No
Cardiocondyla obscurior	Ant	JADYXP010000024.1	2687357-2687431	Abaecin-2	Translated BLASTn Search Result	No
Cataglyphis hispanica	Ant	SGAY01002453.1	10768-10866	Abaecin-2	Translated BLASTn Search Result	No
Cataglyphis niger	Ant	SJPC01000773.1	43935-44033	Abaecin-2	Translated BLASTn Search Result	No
Cyphomyrmex costatus	Ant	LKEX01018418.1	22683-22781	Abaecin-2	Translated BLASTn Search Result	No
Cyphomyrmex costatus	Ant	KYM99867.1	5-75	Abaecin-2	PSI-BLAST Search Result	No
Formica aquilonia x Formica polyctena	Ant	CAJQTV010000019.1	337959-338093	Abaecin-2	Translated BLASTn Search Result	No
Formica exsecta	Ant	NPMM01010057.1	4705229-4705363	Abaecin-2	Translated BLASTn Search Result	No
Formica selysi	Ant	WHNR01000001.1	7214610-7214744	Abaecin-2	Translated BLASTn Search Result	No
Harpagoxenus sublaevis	Ant	JASTWR010000243.1	232292-232390	Abaecin-2	Translated BLASTn Search Result	No
Lasius fuliginosus	Ant	CASCKF010000903.1	58219-58356	Abaecin-2	Translated BLASTn Search Result	No
Lasius niger	Ant	LBMM01010681.1	809-925	Abaecin-2	Translated BLASTn Search Result	No
Monomorium pharaonis	Ant	BBSX02005052.1	8724-8834	Abaecin-2	Translated BLASTn Search Result	No
Mycetomoellerius zeteki	Ant	LKFA01009785.1	2530-2628	Abaecin-2	Translated BLASTn Search Result	Yes
Mycetomoellerius zeteki	Ant	XP 018307440.1	1-54	Abaecin-2	PSI-BLAST Search Result	Yes
Odontomachus brunneus	Ant	JAAGEC010019914.1	6890-7054	Abaecin-2	Translated BLASTn Search Result	No
Pararachymyrmex cornetzi	Ant	LKEY01038114.1	13348-13446	Abaecin-2	Translated BLASTn Search Result	No
Paratrachymyrmex cornetzi	Ant	KYN17872.1	1-54	Abaecin-2	PSI-BLAST Search Result	No
Pogonomyrmex barbatus	Ant	ADIH01028606.1	20225-20383	Abaecin-2	Translated BLASTn Search Result	Yes
Pogonomyrmex barbatus	Ant	XP 025074442.1	1-76	Abaecin-2	PSI-BLAST Search Result	Yes
Pogonomyrmex californicus	Ant	JAEVLP010000124.1	241705-241866	Abaecin-2	Translated BLASTn Search Result	No
Polyergus mexicanus	Ant	JAUDSZ010000018.1	6269843-6269911	Abaecin-2	Translated BLASTn Search Result	No
Prenolepis imparis	Ant	JAUDTA010000006.1	1132366-1132497	Abaecin-2	Translated BLASTn Search Result	No
Pseudoatta argentina	Ant	JAANIA010000071.1	8910073-8910171	Abaecin-2	Translated BLASTn Search Result	No
Pseudomyrmex concolor	Ant	QVOB01009298.1	29470-29565	Abaecin-2	Translated BLASTn Search Result	No
Pseudomyrmex cubaensis	Ant	QVOA01005862.1	11586-11684	Abaecin-2	Translated BLASTn Search Result	No
Pseudomyrmex dendroicus	Ant	QVNZ01010503.1	54-152	Abaecin-2	Translated BLASTn Search Result	No
Pseudomyrmex elongatus	Ant	QVNY01022936.1	5180-5278	Abaecin-2	Translated BLASTn Search Result	No
Pseudomyrmex flavicornis	Ant	QVNW01005555.1	183-278	Abaecin-2	Translated BLASTn Search Result	No
Pseudomyrmex gracilis	Ant	XP 020280064.1	16-78	Abaecin-2	Translated BLASTn Search Result	Yes
Pseudomyrmex gracilis	Ant	JWHX01000040.1	522150-522248	Abaecin-2	PSI-BLAST Search Result	Yes

Pseudomyrmex pallidus	Ant	QVNV01002352.1	85602-85697	Abaecin-2	Translated BLASTn Search Result	No
Pseudomyrmex sp.	Ant	QVNX01019664.1	13231-13329	Abaecin-2	Translated BLASTn Search Result	No
Solenopsis invicta	Ant	PRJNA49629	-	Abaecin-2	BioProject via Zhang and Zhu (2012)	No
Temnothorax americanus	Ant	JASTWO010000091.1	223668-223766	Abaecin-2	Translated BLASTn Search Result	No
Temnothorax curvispinosus	Ant	QBEX01004092.1	321524-321619	Abaecin-2	Translated BLASTn Search Result	No
Temnothorax longispinosus	Ant	QBLH01000329.1	217005-217100	Abaecin-2	Translated BLASTn Search Result	No
Temnothorax ravouxi	Ant	JASTWP010000268.1	128869-128967	Abaecin-2	Translated BLASTn Search Result	No
Temnothorax unifasciatus	Ant	JASTWK010000092.1	127904-127999	Abaecin-2	Translated BLASTn Search Result	No
Tetramorium alpestre	Ant	JAJVSH010000150.1	315640-315738	Abaecin-2	Translated BLASTn Search Result	No
Tetramorium bicarinatum	Ant	VBVP01000517.1	25868-25966	Abaecin-2	Translated BLASTn Search Result	No
Tetramorium immigrans	Ant	VBVO01002714.1	11986-12084	Abaecin-2	Translated BLASTn Search Result	No
Tetramorium parvispinum	Ant	VBVR01000103.1	5246-5344	Abaecin-2	Translated BLASTn Search Result	No
Tetramorium simillimum	Ant	VBVQ01004268.1	8266-8364	Abaecin-2	Translated BLASTn Search Result	No
Trachymyrmex septentrionalis	Ant	LKEZ01022966.1	6697-6765	Abaecin-2	Translated BLASTn Search Result	No
Vollenhovia emeryi	Ant	BBUO01005684.1	5647-5775	Abaecin-2	Translated BLASTn Search Result	No
Wasmannia auropunctata	Ant	BBSV01098925.1	3288-3386	Abaecin-2	Translated BLASTn Search Result	Yes
Wasmannia auropunctata	Ant	XP 011698602.1	1-54	Abaecin-2	PSI-BLAST Search Result	Yes
Mischocyttarus mexicanus	Wasp	KAI4503845.1	1-49	Abaecin-2	Translated BLASTn Search Result	No
Vespula germanica	Wasp	KAF7405905.1	59-112	Abaecin-2	Translated BLASTn Search Result	No
Vespula pensylvanica	Wasp	KAF7429374.1	1-52	Abaecin-2	Translated BLASTn Search Result	No
Vespula vulgaris	Wasp	KAF7402541.1	1-49	Abaecin-2	Translated BLASTn Search Result	No
Solenopsis invicta	Ant	AEAQ02065247.1	1297704-1297841	Abaecin-2	Translated BLASTn search result	No

* Sequences from chromosomes may not include sequence from the shorter second exon, as these are not sufficient to generate BLAST hits

Table S3. ICP analysis sample metadata

					Number of samples used in ICP analysis		
**Colony**	**Species**	**Collection area**	**Colony location (latitude, longitude)**	**Date collected**	**Worker ants**	**Fungus garden**	**Soil**
JKH000424	*Atta texana*	Clear Creek Wildlife Management Area, Louisiana	(31.051, -93.401)*	2021-05-19	1	0	0
JKH000435	*Atta texana*	Clear Creek Wildlife Management Area, Louisiana	(31.05082, -93.40154)	2021-05-22	1	2	2
JKH000423	*Trachymyrmex septentrionalis*	Clear Creek Wildlife Management Area, Louisiana	(31.051, -93.401)*	2021-05-19	1	1	2
JKH000425	*Trachymyrmex septentrionalis*	Alexandria State Forest, Louisiana	(31.11285, -92.4619)	2021-05-21	1	1	2
JKH000426	*Trachymyrmex septentrionalis*	Alexandria State Forest, Louisiana	(31.11294, -92.46684)	2021-05-21	1	1	3
JKH000427	*Trachymyrmex septentrionalis*	Alexandria State Forest, Louisiana	(31.11302, -92.46691)	2021-05-21	1	0	2
JKH000428	*Trachymyrmex septentrionalis*	Alexandria State Forest, Louisiana	(31.11287, -92.4668)	2021-05-21	1	2	1
JKH000429	*Trachymyrmex septentrionalis*	Alexandria State Forest, Louisiana	(31.11262, -92.46777)	2021-05-21	1	0	1
JKH000430	*Trachymyrmex septentrionalis*	Alexandria State Forest, Louisiana	(31.11292, -92.46778)	2021-05-21	1	1	2
JKH000431	*Trachymyrmex septentrionalis*	Alexandria State Forest, Louisiana	(31.11303, -92.46766)	2021-05-21	1	0	2
JKH000432	*Trachymyrmex septentrionalis*	Alexandria State Forest, Louisiana	(31.11284, -92.46739)	2021-05-21	1	1	2

JKH000433	*Trachymyrmex septentrionalis*	Alexandria State Forest, Louisiana	(31.11262, -92.46718)	2021-05-21	1	0	2
JKH000434	*Trachymyrmex septentrionalis*	Alexandria State Forest, Louisiana	(31.11275, -92.46721)	2021-05-21	1	1	2

* Provided coordinates are estimates

Table S4. Susceptibility of *E. coli* JW 0473-3 (Δ*copA*) to *A. texana* and *A. colombica* abaecin-2 variants in combination with the pore-forming peptide cecropin A. All assays were conducted in M9 media.

	***E. coli* JW 0473-3 (Δ*copA*)**				
	MIC_A	MIC_B	MIC_{combo}	FIC Index	Interpretation
Abaecin-2 WT AT + CecA	>128	1	8/0.125	0.31	Synergy
Abaecin-2 H3A AT + CecA	>128	1	8/0.125	0.31	Synergy
Abaecin-2 WT AC + CecA	>128	1	16/0.25	0.31	Synergy
Abaecin-2 H3A AC + CecA	>128	1	8/0.125	0.31	Synergy

Supplementary code S1. Sample DynaFit scripts used for Cu(II) binding affinity determination of abaecin-2 peptides.

A. Sample script for the competition between WT abaecin-2 (12.8 μM) and DP4 (2 μM), assuming 1:1 binding stoichiometry to each abaecin-2 and DP4.

```
; M = Cu
; L = DP2, DP3, or DP4
; P = WT or H3A peptide

[task]
task = fit
data = equilibria

[mechanism]
M + L <==> ML     :  KdL  dissociation
M + P <==> MP     :  KdP  dissociation

[constants] micromolar
KdL = 7.94328e-9
KdP = 1e-7?

[concentrations] micromolar
L = 2
P = 12.8

[responses] F550norm
L = 0.5

[data]
variable     M
set          Data

[output]
directory ./WT+DP4/

[set:Data]
M     F550norm
```

B. Sample script for the competition between WT abaecin-2 (3.6 μM) and DP3 (4 μM), assuming 1:1 binding stoichiometry to each abaecin-2 and DP3.

```
; M = Cu
; L = DP3
; P = WT abaecin-2

[task]
```

```
task = fit
data = equilibria

[mechanism]
M + L <==> ML    :  KdL  dissociation
M + P <==> MP    :  KdP  dissociation

[constants] micromolar
KdL = 5.01187e-7
KdP = 1e-6?

[concentrations] micromolar
L = 4
P = 3.6

[responses] F550
L  = 0.25

[data]
variable   M
set        Data

[output]
directory ./WT+DP3/

[set:Data]
M     F550nm
```

C. Sample script for the competition between H3A abaecin-2 (12.8 μM) and DP2 (2 μM), assuming 1:1 binding stoichiometry to each abaecin-2 and DP2.

```
; M = Cu
; L = DP2
; P = H3A abaecin-2

[task]
task = fit
data = equilibria

[mechanism]
M + L <==> ML    :  KdL  dissociation
M + P <==> MP    :  KdP  dissociation

[constants] micromolar
KdL = 7.94328E-5
KdP = 1e-5?
```

```
[concentrations] micromolar
L = 2
P = 12.8

[responses] F550
L  = 0.5

[data]
variable   M
set        Data

[output]
directory ./H3A+DP2/

[set:Data]
M    F550nm
```
